# Identification of stress-related genes by co-expression network analysis based on the improved turbot genome

**DOI:** 10.1038/s41597-022-01458-4

**Published:** 2022-06-29

**Authors:** Xi-wen Xu, Weiwei Zheng, Zhen Meng, Wenteng Xu, Yingjie Liu, Songlin Chen

**Affiliations:** 1 Laboratory for Marine Fisheries Science and Food Production Processes, Qingdao National Laboratory for Marine Science and Technology, Yellow Sea Fisheries Research Institute, Chinese Academy of Fishery Sciences, Nanjing Road 106, Qingdao, 266071 China; 2 Key Lab of Sustainable Development of Marine Fisheries, Ministry of Agriculture, Wenhai Road 1, Qingdao, 266071 China; 3 College of Fisheries and Life Science, Shanghai Ocean University, Shanghai, China; 4 Chinese Academy of Fishery Sciences, CAFS, 150 Qingta, Yongdinglu-nan, Beijing, 100141 China

**Keywords:** Gene expression profiling, Gene expression, Transcriptomics

## Abstract

Turbot (*Scophthalmus maximus*), commercially important flatfish species, is widely cultivated in Europe and China. With the continuous expansion of the intensive breeding scale, turbot is exposed to various stresses, which greatly impedes the healthy development of turbot industry. Here, we present an improved high-quality chromosome-scale genome assembly of turbot using a combination of PacBio long-read and Illumina short-read sequencing technologies. The genome assembly spans 538.22 Mb comprising 27 contigs with a contig N50 size of 25.76 Mb. Annotation of the genome assembly identified 104.45 Mb repetitive sequences, 22,442 protein-coding genes and 3,345 ncRNAs. Moreover, a total of 345 stress responsive candidate genes were identified by gene co-expression network analysis based on 14 published stress-related RNA-seq datasets consisting of 165 samples. Significantly improved genome assembly and stress-related candidate gene pool will provide valuable resources for further research on turbot functional genome and stress response mechanism, as well as theoretical support for the development of molecular breeding technology for resistant turbot varieties.

## Abstract (summary)

**Table:** 

Measurement(s)	whole genome sequencing
Technology Type(s)	PacBio long-read and Illumina short-read sequencing technologies

## Background & Summary

*Scophthalmus maximus* (FishBase ID: 1348), as known as turbot, an economically important flatfish (Pleuronectiformes), is native to Northeast Atlantic throughout the Mediterranean and along the European coasts to Arctic Circle^1^, and now is the most widely cultivated commercial flatfish around the world with the highest annual aquaculture production^1,2^. Since its firstly introduction into China in 1992, turbot aquaculture industry has made great progress, leading to the rise of the fourth wave of mariculture industry in China^2^. However, turbot was affected by various biotic and abiotic stresses during the breeding process, which seriously threatened the healthy development of turbot aquaculture industry and caused huge economic losses. Therefore, carrying out research on the resistance of turbot and obtaining genetic resources related to stress resistance will contribute to the research on the resistance molecular mechanism of turbot and provide theoretical support for the subsequent genetic improvement of turbot germplasm.

In recent years, numerous RNA-seq studies have been conducted to explore the stress responsive genes and molecular mechanisms under various stresses, such as pathogens stress (*Enteromyxum scophthalmi*^3,4^, *Vibrio anguillarum*^5^), heat stress^6^, oxygen stress^7^, crowding stress^8^, salinity stress^9^, and feeding stress^10^. All these researches were solely focused on the identification of differentially expressed genes (DEGs), whereas connectivity analysis has not yet been taken into account. Instead of focusing only on DEGs, gene co-expression network (GCN) analysis provides new insight into the identification of co-expressed gene modules, their correlation with specific traits, and the pinpointing of key hub genes^11,12^, which cannot be detected by standard transcriptome analysis. This powerful approach has been widely applied to detect diverse stresses response in *Nibea albiflora*^13^, Oysters^14^, *Scophthalmus maximus*^6^, etc.

In this study, we reported an improved high-quality chromosome-scale genome assembly of turbot combing PacBio single molecule sequencing technique (SMRT) and Illumina short-read sequencing technologies. Based on this improved genome assembly, we re-annotated the protein-coding genes, repetitive sequences and ncRNAs. In addition, we re-analyzed multiple stress-related RNA-seq datasets from National Center for Biotechnology Information (NCBI) Sequence Read Archive (SRA) database by gene co-expression network analysis, and identified multiple gene modules and candidate genes response to various stresses in turbot. Taken together, these resources will not only serve as key resources for studying genomics and further research into the stress response mechanisms, but will also promote the progress of genetic improvement and comprehensive stress-resistant molecular breeding of turbot.

## Methods

### Turbot samples and genome sequencing

Genomic DNA was extracted from the muscle samples of a super-female (WW) turbot using Puregene Tissue Core Kit A (Qiagen, USA) according to the manufacturer’s instruction. The quality of the extracted genomic DNA was checked using electrophoresis on 1% agarose gel and the concentration was quantified using a NanoDrop 2000 to ensure the DNA samples met libraries sequencing requirements.

The extracted DNA molecules were firstly used to construct an Illumina pair-end (PE) library with 350 bp insert size using standard protocols provided by Illumina (San Diego, CA, USA). The PE library was then sequenced using the Illumina HiSeq 4000 platform with 150 bp PE mode according to the manufacturer’s instructions. Finally, a total of 51.80 Gb raw reads, accounting ~90X coverage of whole genome, were generated (Table 1).

**Table 1:** Data statistics of whole genome sequencing reads of *S. maximus*.

Library Type	Sequencing Platform	Insert Size (bp)	Raw data (Gb)	Sequence coverage (X)
Illumina	Illumina HiSeq 4000	350	51.80	90
Pacbio	PacBio Sequel II	20,000	150.30	265

We also constructed a 20 kb PacBio library following the PacBio manufacturing protocols (Pacific Biosciences, CA, USA) and sequenced it using the PacBio Sequel II platform with the continuous long-read (CLR) mode following the manufacturer’s instruction. In total, we obtained 150.30 Gb (~265X) PacBio long reads (Table 1). The average and N50 lengths of the subreads were 14.13 kb and 25.47 kb, respectively.

### Genome assembly

**Fig. 1:**
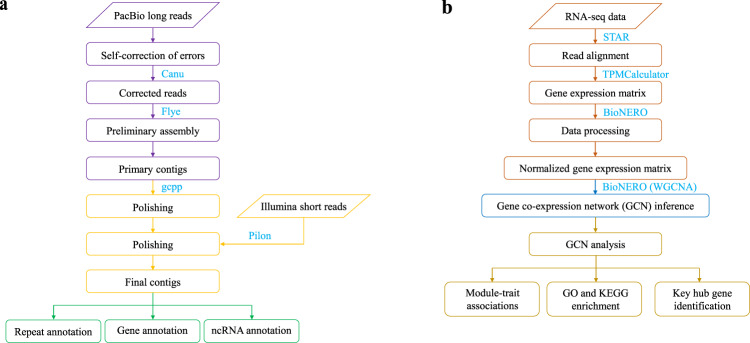
The workflows of genome assembly and gene co-expression network inference used in this study. (**a**) The genome assembly and annotation pipeline. (**b**) The gene co-expression network inference and analyses pipeline.

Long reads generated from the PacBio Sequel II platform were firstly processed by a self-correction of errors using Canu^15^ with default parameters. And then corrected reads were subsequently assembled by Flye (v2.7)^16^ (--pacbio-corr -- threads 80 --genome-size 568 m). To obtain the final assembly, the draft assembly was removed haplotypic duplication by purge_dups^17^ and polished by gcpp (https://github.com/PacificBiosciences/gcpp) with default parameters using PacBio data, then Pilon^18^(--fix bases) was used to further polish the genome using Illumina data (Fig. 1a). Finally, we obtained a new assembled genome of turbot containing 27 contigs with a total length of 538.22 Mb and a contig N50 length of 25.76 Mb, exhibiting higher contiguity and completeness comparable to other published turbot genomes^19–21^ (Table 2). In addition, GC content of the genome assembly was estimated to be 43.53%.

**Table 2:** Comparative statistic of the *S. maximus* genome assembly with old ones.

Genome assembly	This study	Martínez *et al.*^21^	Xu *et al.*^19^		Figueras *et al.*^20^
			female	male	
Scaffold N50 (Mb)	25.76	25.95	25.17	5.93	24.81
Contig N50 (Mb)	25.76	20.47	0.028	0.045	0.054
Total scaffold number	27	127	28,256	9,724	22
Total contig number	27	178	65,796	36,500	21,326
Total length (Mb)	538.22	556.70	568.47	587.19	524.98
GC Content (%)	43.53	43.30	43.42	43.70	43.30

### Genome annotation

We detected and classified repetitive sequences in the final turbot genome assembly by a combination of homology-based and *de novo* prediction strategies. In homology-based searching, known repeats were identified using RepeatMasker (V4.1.1)^22^ based on the RepBase TE library (version 10/26/2018)^23^. In addition, *de novo* prediction was conducted using RepeatMasker to further detect novel repeats, which based on the *de novo* repeats library of the turbot genome constructed with RepeatModeler (http://www.repeatmasker.org/RepeatModeler/) and LTR-FINDER^24^. Finally, a total of 104.45 Mb of non-redundant repetitive sequences (Combined TEs) were obtained, accounting for 19.41% of the assembled genome (Table 3). Amid predominant repeats, DNA transposons were the most abundant (54.16 Mb), representing 10.06% of the genome, followed by long interspersed elements (LINEs, 3.10%), long terminal repeats (LTRs, 2.95%) and short interspersed nuclear elements (SINEs, 0.51%) (Table 3).

**Table 3:** Classified statistics of repeat sequences of *S. maximus*.

	RepBase TEs		TE Proteins		*De novo*		Combined TEs	
	Length (bp)	% in Genome	Length (bp)	% in Genome	Length (bp)	% in Genome	Length (bp)	% in Genome
DNA	38,217,303	7.10	2,321,886	0.43	23,128,062	4.30	54,159,141	10.06
LINE	13,026,936	2.42	6,871,234	1.28	7,405,321	1.38	16,693,988	3.10
SINE	2,309,601	0.43	0	0	857,212	0.16	2,740,574	0.51
LTR	11,363,027	2.11	2,222,887	0.41	4,790,157	0.89	15,901,294	2.95
Satellite	2,989,136	0.56	0	0	499,041	0.09	3,462,111	0.64
Simple_repeat	0	0	0	0	0	0	0	0
Other	2,814	0	135	0	0	0	2,949	0
Unknown	537,749	0.10	13,890	0	23,176,727	4.31	23,566,810	4.38
Total	58,685,000	10.90	11,419,271	2.12	58,413,629	10.85	104,452,847	19.41

**Fig. 2:**
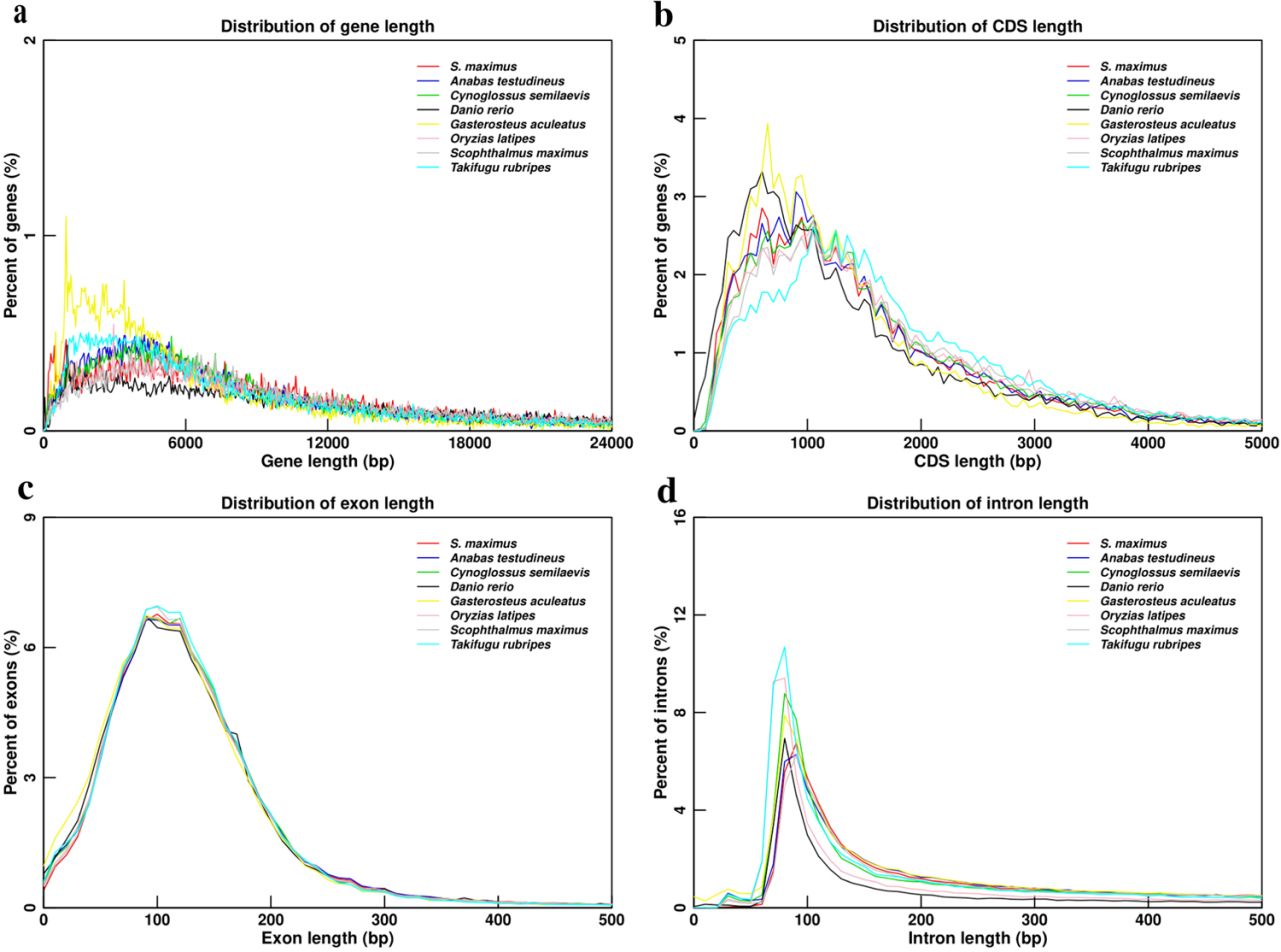
Comparisons of gene features among *S. maximus*, *Anabas testudineus, Cynoglossus semilaevis, Danio rerio, Gasterosteus aculeatus, Oryzias latipes, Scophthalmus maximux* and *Takifugu rubripes*. (**a**) Gene length distributions of the species. (**b**) CDS length distributions of the species. (**c**) Exon length distributions of the species. (**d**) Intron length distributions of the species.

Protein-coding gene annotations were then conducted with MAKER (v3.01.03)^25^ by a combined strategy of homology-based, *de novo*, and transcriptome-assisted predictions. For homology-based prediction, protein sequences of seven teleost species, *Anabas testudineus, Cynoglossus semilaevis, Danio rerio, Gasterosteus aculeatus, Oryzias latipes, Scophthalmus maximux, Takifugu rubripes*, were downloaded from Ensembl and NCBI, and mappped to turbot genome using TBLASTN^26^ (e-value ≤ 1e-5). Exonerate (v2.4.0)^27^ was used to align homologous protein sequences to turbot genome. Homologous genes were predicted ranging from 35,093 to 48,770 in above species reference sequences (Table 4). For *de novo* prediction, Augustus^28^ and Genscan^29^ were employed to analyze the repeats masked genome, which detected 30,320 and 40,007 genes, respectively (Table 4). For transcriptome-assisted prediction, RNA-seq data (NCBI accession number: SRP261889, SRP273870) were aligned to turbot genome to identify potential gene structures, and 16,356 genes were supported. Finally, we performed MAKER (v3.01.03) to integrate genes generated by above predictions to produce a consensus protein-coding gene set consisting of 22,442 genes with an average gene length of 15,828 bp (Table 4). Comparisons of gene features between turbot and other seven species indicated similar distribution patterns in average length of gene, coding sequence (CDS), exon and intron (Fig. 2).

**Table 4:** General statistics of predicted protein-coding genes in *S. maximus* genome.

Gene set		Protein coding gene number	Average gene length (bp)	Average CDS length (bp)	Average exon per gene	Average exon length (bp)	Average intron length (bp)
*De novo*	Genscan	30,320	12,927	1,595	8.92	178.87	1,431
	AUGUSTUS	40,007	8,114	1,220	6.53	186.85	1,246
Homolog	*D.rerio*	38,658	12,345	1,120	6.69	167.55	1,974
	*S.maximus*	40,864	12,956	1,153	6.74	171.11	2,056
	*G.aculeatus*	35,093	11,413	1,114	6.89	161.56	1,748
	*A.testudineus*	39,404	14,059	1,167	6.69	174.59	2,267
	*C.semilaevis*	37,758	12,151	1,163	6.84	169.92	1,880
	*O.latipes*	40,717	14,894	1,149	6.36	180.75	2,566
	*T.rubripes*	48,770	12,386	950.24	5.65	168.14	2,458
trans.orf/RNAseq		16,356	19,894	2,040	12.87	358.55	1,287
MAKER		22,442	15,828	1,703	10.51	327.83	1,302

To obtain functional annotation of the predicted protein-coding genes in turbot genome, InterPro^30^, Pfam^31^, Swissprot^32^ and TrEMBL^32^ databases were respectively used to predict protein function based on the conserved protein domains by InterProScan (v5.46)^33^. BLASTP (e-value ≤ 1e-5) was used for the homolog search in multiple databases, such as Gene Ontology (GO)^34^, Kyoto Encyclopedia of Genes and Genomes (KEGG)^35^, and NCBI non-redundant protein (NR)^36^ databases. Ultimately, a total of 21,360 genes (95.18% of all predicted genes) could be functionally annotated by at least one of the abovementioned databases (Table 5).

**Table 5:** General statistics of gene function annotation of *S. maximus*.

Type		Number	Percent (%)
Total		22,442	
Annotated		21,360	95.18
	InterPro	19,732	87.92
	GO	15,096	67.27
	KEGG_ALL	20,917	93.2
	KEGG_KO	13,810	61.54
	Swissprot	19,137	85.27
	TrEMBL	21,313	94.97
	TF	3,328	14.83
	Pfam	19,126	85.22
	NR	21,065	93.86
	KOG	17,738	79.04
Unannotated		1,082	4.82

For non-coding genes, a total of 1,796 tRNAs were identified using tRNAscan-SE^37^. Moreover, 538 rRNAs were detected through searching for homology against rRNA sequences of related species using BLASTN. Besides, 430 miRNAs and 581 snRNAs were predicted using INFERNAL^38^ tool based on Rfam database (Table 6), respectively.

**Table 6:** General statistics of non-coding annotation of *S. maximus*.

Type		Copy	Average length(bp)	Total length(bp)	% of genome
miRNA		430	85	36,407	0.006764
tRNA		1,796	75	134,264	0.024946
rRNA	rRNA	538	138	74,432	0.013829
	18 S	6	1,849	11,094	0.002061
	28 S	0	0	0	0
	5.8 S	8	156	1,247	0.000232
	5 S	524	118	62,091	0.011536
snRNA	snRNA	581	137	79,403	0.014753
	CD-box	193	121	23,313	0.004332
	HACA-box	75	151	11,302	0.002100
	splicing	306	141	43,069	0.008002
	scaRNA	7	246	1,719	0.000319

### Gene co-expression network inference and module-trait associations analysis

A total of 165 published stress-related RNA-seq samples data from 14 independent SRA studies (Table 7) that surveyed transcriptome profiling in turbot under different stresses (i.e., crowding, feeding, heat, oxygen, pathogens, and salinity) were downloaded from the NCBI SRA database using SRAtoolkit (v2.11.0)^39^. Following, RNA-seq data in SRA format were converted into FASTQ format using fastq-dump tool of SRAtoolkit. Then, reads were aligned to the latest assembled turbot genome using STAR^40^ with default parameters. TPMCalculator (-q 1)^41^ was used to calculate transcripts per million (TPM) values for all genes using sorted bam files obtained from reads alignment. Subsequently, we used BioNERO^42^ to preprocess the gene expression data according to the following steps: I) Replacing missing values (NAs) with 0 using replace_na function; II) Removing the genes whose average gene expression was less than 1 with remove_nonexp function; III) Removing outlying samples with ZKfiltering function; IV) Adjusting for confounding artifacts with PC_correction function to make every gene follow an approximate normal distribution. After filtering and processing (Fig. 1b), a normalized gene expression matrix consisting of 12,271 genes with medial expression value ≥ 1 from 160 RNA-seq samples were obtained.

**Table 7:** Overview of the RNA-seq datasets used in this study.

Stress		SRA Study	SRA-Experiments	Number of individuals	Platform (Illumina)	Size (GB)	References
Crowding	—	SRP129900	12	500	HiSeq 4000	68.20	^8^
Feeding	*myo*-inositol	SRP188583	15	300	HiSeq 4000	115.45	^10^
	fish meal, soybean meal	SRP074811	2	360	NextSeq 500	42.56	^87^
	sodium butyrate, soybean meal	SRP275545	6	270	HiSeq 2000	50.23	^88^
Heat	—	SRP152627	10	—	HiSeq 4000	88.99	^6^
Oxygen	—	SRP167318	9	9	HiSeq 2500	58.99	^7^
Pathogens	*Enteromyxum scophthalmi*	SRP308109	49	280	HiSeq 4000	381.62	^3^
		SRP255305	10	120	HiSeq 4000	17.55	^89^
		SRP065375	12	—	HiSeq 2000	31.48	^4^
		SRP050607	12	120	HiSeq 2000	36.02	^90^
	*Vibrio anguillarum*	SRP191266	4	90	HiSeq 2500	53.34	^5^
Salinity	—	SRP277001	6	360	HiSeq 4000	49.35	^91^
		SRP238143	9	180	HiSeq 2000	70.48	^92^
		SRP153594	9	—	HiSeq 4000	70.86	^9^
Total	—	—	165		—	1135.12	

**Fig. 3:**
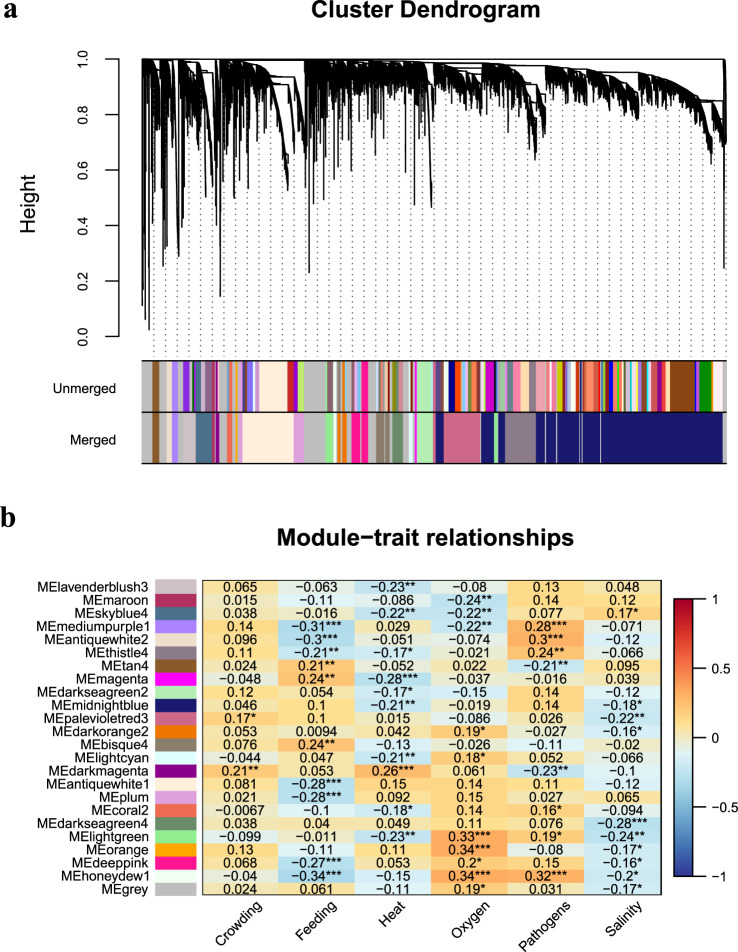
Gene co-expression network analysis of different stresses. (**a**) Cluster Dendrogram of genes and modules. The branches and color bands represent the assigned module. The tips of the branches represent genes. (**b**) Correlation between modules and stresses. The value in the box is the correlation coefficients. Correlation coefficients with ** or *** represent extremely significant correlation and significant correlation with *.

After we filtered and normalized the expression data, BioNERO^42^ was used to construct a gene co-expression network (GCN) (Fig. 1b). First of all, we identified the most optimal β power to make the network satisfy the scale-free topology with the function SFT_fit. According to the result, the optimal power is 11, for which the scale-free topology fit index (R^2^) reaches 0.8 and mean connectivity tends to 0. Next, we used the exp2gcn function to infer the GCN with power 11. As a result, a total of 24 co-expression modules were eventually identified (Fig. 3a), with the number of genes per module ranging from 34 (magenta) to 4,396 (midnightblue).

We then identified modules that were extremely significant (*p*-value < 0.01) positively or negatively correlated with particular traits (stresses) by calculating module-trait spearman correlation coefficients using the module_trait_cor function from BioNERO. As shown in Fig. 3b, significantly related modules could be found for each trait, which provides rich resources for the study of turbot stress resistance mechanism. To detect the functionality of the modules that extremely significant correlated with each stress, for each module, GO and KEGG enrichment analyses were performed on all genes in the module using TBtools^43^ (corrected *p-value* (BH method) < 0.5).

### Identification of key hub genes

To identify candidate key hub genes related to every stress, we firstly constructed hub genes set. Hub genes, defined as the top 10% genes with highest degree (i.e., sum of connection weights of a gene to all other genes in the module) that have module membership (MM) (i.e., correlation of a gene to its module eigengene) > 0.8, were identified using the function get_hubs_gcn. Then, hub genes belonging to modules that were extremely significant associated with same stress were merged as hub genes set for this stress. Following, we set up the differentially expressed genes (DEGs) set. Firstly, we used featureCounts^44^ software program in Subread^45^ package to construct reads count matrixes. Then, edgeR^46^ was used to identify DEGs with false discovery rate (FDR) < 0.05 and |log_2_FC| > 1. DEGs, related to the same stress, were merged as DEGs set for this stress. Finally, genes, included in both hub genes set and DEGs set corresponding to the same stress, were defined as the candidate key hub genes for this stress. Candidate key hub genes related to crowding, feeding, heat, oxygen, pathogens and salinity stress were 0, 128, 40, 7, 90 and 80, respectively.

### Heat-related modules enrichment analysis and identification of key hub genes

To heat stress, GO enrichment analyses illustrated that metabolic process, cellular process, catabolic process, catalytic activity, hydrolase activity, oxidoreductase activity, cellular response to stress, biosynthetic process, and binding were the significantly enriched terms (GO enrichment.xlsx^47^) in modules that were extremely significant correlated with heat stress. Meanwhile, KEGG enrichment analyses were employed on the same modules, and the results manifested that metabolism, lipid metabolism, carbohydrate metabolism, glycolysis/gluconeogenesis, PPAR signaling pathway, proteasome, digestive system, fat digestion and absorption, peroxisome, cell growth and death, transport and catabolism, cellular processes, and protein kinases were the significantly enriched pathways (KEGG enrichment.xlsx^47^). Finally, we identified 40 candidate heat-related key hub genes, of which 7 genes including ABI1^48^, CD44^49^, CCDC153^50^, G2e3^51^, PATJ^52^, HYKK^53^ and occludin^54^ has been verified to contribute to heat stress. For instance, G2e3 was one of the candidate genes in the liver of heat-treated large yellow croaker^51^. Exposure to heat stress (39 °C or 41 °C) resulted in increased expression of occludin protein in Caco-2 cells^54^.

### Oxygen-related modules enrichment analysis and identification of key hub genes

To oxygen stress, GO enrichment results showed that aerobic respiration, aerobic electron transport chain, metabolic process, oxidoreductase activity, mitochondrial inner membrane, mitochondrial respirasome, respiratory chain complex, oxidative phosphorylation, oxidoreductase complex, catabolic process, catalytic activity, response to stress, response to external stimulus were the significantly enriched terms (GO enrichment.xlsx^47^) in modules that were extremely significant correlated with oxygen stress. Furthermore, we employed KEGG enrichment analyses on the same modules, and the results demonstrated that metabolism, oxidative phosphorylation, environmental adaptation, energy metabolism, and peroxisome were significantly enriched pathways (KEGG enrichment.xlsx^47^). In addition, we obtained 7 candidate oxygen-related hub genes, among which AMBP^55^ and CNN1^56^ had been confirmed to conduce to heat stress. Such as, the gene for A1M is denoted AMBP, which has a physiological role as a protective antioxidant^55^. Five percent oxygen concentration significantly increased the expression levels of CNN1 in adipose-derived stem cell cultures after 2 weeks of induction^56^.

### Pathogens-related modules enrichment analysis and identification of key hub genes

To pathogens stress, GO enrichment results showed that immune response, immune system process, response to wound healing, blood coagulation, hemostasis, response to external stimulus, response to stress, biosynthetic process, catalytic activity, metabolic process, and cellular process were the significantly enriched terms (GO enrichment.xlsx^47^) in modules that were extremely significant related with pathogens stress. Meanwhile, KEGG enrichment analyses were employed on the same modules, and the results manifested that immune system, human diseases, complement and coagulation cascades, CD molecules, lysosome, phagosome, B cell receptor signaling pathway, hematopoietic cell lineage metabolism, glycosaminoglycan binding proteins, exosome, neutrophil extracellular trap formation, were the significantly enriched pathways (KEGG enrichment.xlsx^47^). Ultimately, we determined 90 candidate pathogens-relevant hub genes, thereinto, 18 genes, such as CMKLR1^57^, CSF3R^58^, SIGLEC10^59^, RAP1GAP2^60^, Cd300lf^61^, NPTN^62^, MRC1^63^, LILRA6^64^, BLNK^65^, CXCL12^66^, PIGR^67^, SIGLEC15^68^, GULP1^69^, MARCO^70^, NLRP12^71^, CRP^72^, FGG^73^, and lysozyme^74^, had been proved to be conducive to pathogens stress. For example, LILRA6 is essential for macrophage-mediated immune responses and it has the potential to complement the innate and adaptive immune system against pathogens^64^. Siglec-15 probably plays a conserved, regulatory role in the immune system of vertebrates^68^. In addition to its direct antimicrobial role, more recent evidence has shown that lysozyme modulates the host immune response to infection^74^.

### Salinity-related modules enrichment analysis and identification of key hub genes

To salinity stress, according to GO enrichment analyses, terms (GO enrichment.xlsx^47^), such as, ion binding, small molecule binding, anion binding, proteasome complex, proteasome-activating activity, catabolic process, metabolic process, cellular process, cellular response to stress, binding, and ATP binding were significantly enriched in modules that were extremely significant related with salinity stress. Simultaneously, we employed KEGG enrichment analyses on the same modules, and the results indicated that proteasome pathway, protein kinases were the significantly enriched pathways (KEGG enrichment.xlsx^47^). After taking the intersection of hub genes set and DEGs set, 80 genes were defined as candidate salinity-associated hub genes, and six genes (NDUFV1^75^, EMSY^76^, RBBP6^77^, ATF2^78^, Map3k7^79^ and PSMC2^80^) had been certified to be related to salinity stress. For example, RBBP6 was one of the identified candidate genes for freshwater vs. marine adaptation in threespine stickleback^77^. MAP3K7, also known as TAK1, could be highly activated by osmotic stress^79^.

## Data Records

The raw data, including Illumina and PacBio sequencing data of the whole genome, was submitted to the NCBI SRA with accession number SRP352610^81^. The final genome assembly and annotation gff file are available at National Genomics Data Center with accession number GWHBHEA00000000.1^82^. The final genome assembly is also available through NCBI with accession number GCA_022379125.1^83^. The functional annotation of protein-coding genes, gene expression matrix used for gene co-expression network inference, and gene co-expression network analysis results including genes per module, hub genes set, DEGs set, GO and KEGG enrichment, key hub genes, are available at Figshare^47^.

## Technical Validation

### Quality and completeness assessment of genome assembly

The quality and completeness of the new assembly were evaluated through three independent approaches. Firstly, the base-level accuracy and completeness were estimated using Merqury^84^ by comparing k-mers in the assembly to those found in the high-accuracy Illumina reads. The results revealed that per-base accuracy rates for turbot assembly was 0.999994 and completeness value was 99.38%. Secondly, the completeness of the final assembled genome was also assessed using Benchmarking Universal Single-Copy Orthologs (BUSCO v4.1.6)^85^ with 4,584 single-copy orthologs from actinopterygii_odb9 database. BUSCO analysis revealed that 97.4% (4,465) complete BUSCOs (94.9% single-copy and 2.5% duplicated BUSCOs) and 1.1% (49) fragmented BUSCOs were identified in the assembled genome of turbot. Thirdly, we further evaluated the assembly quality using Inspector^86^ by aligning PacBio long reads to the assembled contigs for generating read-to-contig alignment and performing downstream assembly evaluation. As a result, read-to-contig mapping rate and quality value (QV) were 91.93% and 45.41, respectively. All these indicators suggested a high-quality and complete genome assembly for the further research in genetics and genomics of turbot.

## Data Availability

The data analysis methods, software and associated parameters used in this study are described in the Methods section. Default parameters were applied if no parameter was described. No custom scripts were generated in this work.
